# Forever young: SIRT3 a shield against mitochondrial meltdown, aging, and neurodegeneration

**DOI:** 10.3389/fnagi.2013.00048

**Published:** 2013-09-06

**Authors:** Brad Kincaid, Ella Bossy-Wetzel

**Affiliations:** Burnett School of Biomedical Sciences, College of Medicine, University of Central FloridaOrlando, FL, USA

**Keywords:** SIRT3, neuroprotection, caloric restriction, aging, neurodegeneration, antioxidants, mitochondria

## Abstract

Caloric restriction (CR), fasting, and exercise have long been recognized for their neuroprotective and lifespan-extending properties; however, the underlying mechanisms of these phenomena remain elusive. Such extraordinary benefits might be linked to the activation of sirtuins. In mammals, the sirtuin family has seven members (SIRT1–7), which diverge in tissue distribution, subcellular localization, enzymatic activity, and targets. SIRT1, SIRT2, and SIRT3 have deacetylase activity. Their dependence on NAD^+^ directly links their activity to the metabolic status of the cell. High NAD^+^ levels convey neuroprotective effects, possibly via activation of sirtuin family members. Mitochondrial sirtuin 3 (SIRT3) has received much attention for its role in metabolism and aging. Specific small nucleotide polymorphisms in *Sirt3* are linked to increased human lifespan. SIRT3 mediates the adaptation of increased energy demand during CR, fasting, and exercise to increased production of energy equivalents. SIRT3 deacetylates and activates mitochondrial enzymes involved in fatty acid β-oxidation, amino acid metabolism, the electron transport chain, and antioxidant defenses. As a result, the mitochondrial energy metabolism increases. In addition, SIRT3 prevents apoptosis by lowering reactive oxygen species and inhibiting components of the mitochondrial permeability transition pore. Mitochondrial deficits associated with aging and neurodegeneration might therefore be slowed or even prevented by SIRT3 activation. In addition, upregulating SIRT3 activity by dietary supplementation of sirtuin activating compounds might promote the beneficial effects of this enzyme. The goal of this review is to summarize emerging data supporting a neuroprotective action of SIRT3 against Alzheimer’s disease, Huntington’s disease, Parkinson’s disease, and amyotrophic lateral sclerosis.

## INTRODUCTION

Caloric restriction (CR), fasting, and exercise promote neuroprotection and extend healthy lifespan in mammals. Reducing food consumption without malnutrition extends the lifespan of rodents by up to 50% (Weindruch et al., 1986; McCay et al., 1989). Recent studies suggested these extraordinary benefits may be linked to upregulation of sirtuins.

Sirtuins were first described as NAD^+^-dependent type III histone deacetylases with yeast Sir2 as its founding member, silencing gene expression by histone deacetylation (Guarente and Kenyon, 2000). However, mammalian sirtuins target not only histones in the nucleus but also other proteins in the cytoplasm and mitochondria. In mammals, the sirtuin family has seven members (SIRT1–7), which differ in tissue distribution, subcellular localization, enzymatic activity, and target proteins. SIRT1, SIRT6, and SIRT7 are present in the nucleus; SIRT2 in the cytoplasm; and SIRT3, SIRT4, and SIRT5 in mitochondria (Frye, 2000). Based on sequence homology, SIRT1, SIRT2, and SIRT3 belong to class I sirtuins and exhibit deacetylase activity. SIRT4 belongs to class II and has ADP-ribosylation activity; SIRT5 to class III and has demalonylation and desuccinylation activity; and SIRT6 and SIRT7 to class IV (He et al., 2012). SIRT6 has deacetylase and ADP-ribosylase activity, while SIRT7 has no reported activity.

Sirtuin enzymatic activity requires NAD^+^ as a cofactor whose levels increase by energy stress which occurs, e.g., during fasting, CR, and exercise. Thus, NAD^+^ mediates the adaptive response to low energy by activating sirtuins and their downstream targets. Sirtuins transform NAD^+^ to nicotinamide, which acts as a competitive inhibitor of sirtuins by a negative feedback mechanism. The other breakdown product of NAD^+^ is *O*-acetyl-ADP-ribose.

The average lifespan of humans has steadily increased as a result of advances in medicine and improved living conditions. Despite this progress, the maximum human lifespan remains constant, for reasons not entirely clear. Among the aging theories is the “free radical theory of aging” proposed by Denham Harman in the 1950s, which attempts to explain the limit on human lifespan as the result of accumulated damage to proteins, nucleic acids, lipids, and organelles by free radicals (Harman, 1956; Weissman et al., 2007; Wong and Cuervo, 2010). Mitochondria are both the source and target of reactive oxygen species (ROS) in cells including superoxide anions, hydrogen peroxide, hydroxyl radicals, and reactive nitrogen species such as peroxynitrite. Oxidative and nitrosative stress can evoke irreversible damage to proteins, lipids, and DNA. As a result, mitochondria have been linked to aging and age-related diseases (Hall et al., 2001; Singh, 2006). SIRT3 emerged as a protein of particular interest to the aging field due to its mitochondrial localization and association with exceptional long lifespan in humans (Hurst et al., 2002). SIRT3 deacetylates and activates many mitochondrial enzymes involved in fatty acid β-oxidation, amino acid metabolism, the electron transport chain (ETC), and antioxidant defenses. Neurons are especially sensitive to insults that result in energy depletion and oxidative stress (Du et al., 2003). Here, we will review the well-documented roles of SIRT3 in metabolism and antioxidant defenses, and the new evidence linking SIRT3 to neuroprotection.

## SIRT3 SUBCELLULAR LOCALIZATION

Although most reports indicated an exclusive mitochondrial localization of SIRT3 (Onyango et al., 2002; Shi et al., 2005; Schwer et al., 2006; Cooper and Spelbrink, 2008; Gurd et al., 2012), others have argued that SIRT3 is also present in the nucleus and cytoplasm (Scher et al., 2007; Sundaresan et al., 2008; Shulga et al., 2010; Iwahara et al., 2012). SIRT3 target proteins were identified in all three compartments: nucleus, cytoplasm, and mitochondrion (He et al., 2012). Thus, the localization and role of SIRT3 in different cellular compartments is a matter of considerable debate.

Within mitochondria, SIRT3 appears to be localized to the inner mitochondrial membrane cristae and the matrix (Shi et al., 2005; Schwer et al., 2006). Additional investigations are needed to determine whether SIRT3 also targets to the outer mitochondrial membrane or the intermembrane space.

## SIRT3 ISOFORMS

Initial reports indicated that human SIRT3 is present in a full-length form and short form. Full-length SIRT3 translocates from the cytoplasm to mitochondria. Once imported into mitochondria, SIRT3 is processed to the short form (Schwer et al., 2006). Initially only the short, mitochondrial form of human SIRT3 was thought to exhibit NAD^+^-dependent deacetylase activity (Schwer et al., 2006). However, several reports challenged this view and demonstrated that both the full-length and short form of SIRT3 exhibit deacetylase activity. In agreement, full-length SIRT3 accumulates in the nucleus and deacetylates histones (Lombard et al., 2007). In cardiomyocytes, full-length mouse SIRT3 is present in the nucleus and cytoplasm, while the short form is only found in mitochondria (Sundaresan et al., 2008). SIRT3 deacetylates Ku-70 in the cytoplasm and thereby prevents pro-apoptotic BAX to translocate to mitochondria. In addition, three different splice variants of mouse SIRT3 were discovered (Jin et al., 2009). Two of these splice variants contain an amino-terminal mitochondrial targeting sequence (MTS); however, the third form lacks the MTS (Cooper et al., 2009; Jin et al., 2009). Upon translocation to mitochondria, the MTS is cleaved by a matrix processing peptidase to produce a short form of SIRT3. Without the MTS, SIRT3 fails to translocate to mitochondria (Schwer et al., 2002).

## *Sirt*3 POLYMORPHISMS

Initial interest in SIRT3 and its role in human aging was sparked by the discovery of unique single nucleotide polymorphisms (SNPs) linked to centenarians (Hurst et al., 2002; Rose et al., 2003; Bellizzi et al., 2005). Males carrying the G477T transversion in exon 3 of *Sirt3* are healthy and live beyond the average lifespan. However, this nucleotide transition is silent and does not alter the amino acid sequence (Rose et al., 2003). Therefore, the mechanism by which this SNP prolongs life is unknown.

The second SNP involves the variable number tandem repeat (VNTR) region within intron 5 of *Sirt3* (Bellizzi et al., 2005). The specific VNTR polymorphism exhibits improved enhancer activity and increases *Sirt3* expression (Bellizzi et al., 2005). Interestingly, a strong linkage disequilibrium has been observed between the VNTR and G477T polymorphism (Bellizzi et al., 2005). Thus, one can speculate that the two polymorphisms might exhibit cooperative effects. Of additional note, *Sirt3* maps to a region within the short arm of chromosome 11 shared by a cluster of longevity genes, including insulin-like growth factor 2 (*IGF2*), proinsulin (*INS*), and tyrosine hydroxylase (*TH*). Therefore, it is possible that *Sirt3* shares common regulatory elements with these genes (Hurst et al., 2002).

Finally, a third SNP in the coding sequence of *Sirt3* has been discovered. Unlike the first two SNPs that are linked to increased lifespan, the third SNP increases the risk for age-related metabolic syndrome. The particular SNP involves an amino acid substitution in the conserved deacetylase catalytic region, resulting in lower SIRT3 deacetylase activity (Hirschey et al., 2011). Taken together, additional studies are required to investigate how the various SNPs modify human health and lifespan.

## SIRT3 TISSUE-SPECIFIC EXPRESSION

SIRT3 expression is highest in metabolically active tissues including the brain, heart, liver, brown adipose tissue (BAT), and skeletal muscle (Lombard et al., 2007; Ahn et al., 2008; Gurd et al., 2012). SIRT3 knockout (KO) mice show no obvious phenotypic changes, but are prone to age-linked diseases including metabolic syndrome, cancer, and cardiac failure (Hirschey et al., 2011). Whether SIRT3 KO mice exhibit neurological defects has yet to be elucidated. SIRT3 deletion in mice causes hyperacetylation of mitochondrial proteins and major metabolic defects. Mass spectrometry revealed that 65% of all proteins in mitochondria have at least one lysine acetylated in liver tissue of SIRT3 KO mice (Hebert et al., 2013). By contrast, SIRT4 KO and SIRT5 KO mice do not exhibit global hyperacetylation of mitochondrial proteins (Lombard et al., 2007). In fact, SIRT4 and SIRT5 have either no or only very weak NAD^+^-dependent deacetylase activity (Verdin et al., 2010). SIRT4 has ADP-ribosylase activity and SIRT5 has demalonylase and desuccinylase activities (Haigis et al., 2006; Du et al., 2011). Therefore, SIRT3 is the key deacetylase in mitochondria. Future studies using conditional, tissue-specific knock-in or knockout mice will establish the functions of SIRT3 in tissues such as the brain.

## MITOCHONDRIAL DYNAMICS

Energy homeostasis is a delicate balance between energy supply, use, and storage. Mitochondria are highly dynamic organelles and undergo constant cycles of fission and fusion. This dynamic process is modified by nutrient supply, linking cellular energy production with energy demands. For example, nutrient excess stimulates mitochondrial fission, fragmented mitochondrial morphology, and an arrest in oxygen consumption, oxidative phosphorylation, and ATP synthesis. By contrast, nutrient deficiency triggers mitochondrial fusion, elongated mitochondrial morphology, and an acceleration of mitochondrial respiration and ATP production (Molina et al., 2009; Gomes and Scorrano, 2011; Rambold et al., 2011; Liesa and Shirihai, 2013). Thus, mitochondrial fission and fusion is a fine tuned process that controls the switches of energy production with energy demand, thereby maintaining homeostasis.

Mitochondrial fission and fusion is regulated by conserved large GTPases of the Dynamin family, which exert opposite functions. Fission is mediated by dynamin related protein 1 (DRP1) while fusion is controlled by optic atrophy 1 (OPA1), mitofusin 1 (MFN1), and mitofusin 2 (MFN2; Arnoult et al., 2005; Misko et al., 2010). It can be speculated whether increased SIRT1 and SIRT3 activity during CR, fasting, and exercise enhance energy metabolism by activating fusion and inhibiting fission of mitochondria. In support, sirtuin activating compounds, e.g., NAD^+^ and resveratrol, seem to promote a fused mitochondrial morphology. In addition, exercise promotes DRP1 phosphorylation at serine 637 by protein kinase A (PKA), a process thought to inhibit DRP1 function and decrease mitochondrial fission. Future studies are needed to investigate how mitochondrial fission/fusion GTPases are regulated by dietary changes and sirtuins.

Mitochondrial dynamics is also implicated in aging. For example, reducing mitochondrial fission by DRP1 deletion slows aging and increases lifespan of yeast (Scheckhuber et al., 2007). Similarly, DRP1 inactivation in worm enhances the positive effects of reduced insulin signaling on lifespan extension (Yang et al., 2011). Thus, promoting mitochondrial fusion correlates with longevity of these model organisms. Whether mitochondrial dynamics regulates the aging process in mammals remains to be investigated.

A balance between mitochondrial fission and fusion plays a critical role in neuronal function and survival (Knott et al., 2008). In neurons, mitochondrial dynamics is required for bioenergetic and synaptic function, Ca^2^^+^ buffering, transport of mitochondria across neurites, removal of damaged mitochondria by autophagy, and neuronal cell survival. Future studies are needed to investigate whether SIRT3 enhances neuronal survival by regulating processes such as mitochondrial fission and fusion.

## SIRT3 IN CR AND EXERCISE

More than 70 years ago the lifespan-extending properties of a CR diet were demonstrated in rats (McCay et al., 1989). The details of this observation were not further explored until almost half a century later when Walford and Weindruch discovered that reducing food consumption 25–60% without malnutrition extends the lifespan of mice up to 50% (Weindruch et al., 1986). More recently, CR has been shown to inhibit neurodegeneration in animal models of both Alzheimer’s disease (AD) and Parkinson’s disease (PD; Zhu et al., 1999; Mattson, 2000). A deeper look into the metabolic and cellular changes that occur during metabolic stresses such as CR may reveal the unique mechanisms by which these benefits are conferred.

During the transition from the fed to fasted state, blood glucose levels decline causing carbohydrate utilization and fatty acid synthesis to cease in the liver, while fatty acid oxidation and ketogenesis are induced (McGarry and Foster, 1980). Furthermore, prolonged starvation results in increased amino acid catabolism as cells search for a new source of energy. Autophagy is also increased during nutrient stress (Kuma et al., 2004). Blocking autophagy accelerates cell death, aging, and neurodegeneration (Kuma et al., 2004; Hara et al., 2006; Komatsu et al., 2006). Interestingly, NAD^+^ levels greatly increase during CR in muscle and white adipose tissue (WAT), thus suggesting potential upregulation of Sirtuin activity (Chen et al., 2008).

Remarkably, SIRT3 expression levels fluctuate with diet. Numerous studies have reported CR-induced increased SIRT3 expression (Palacios et al., 2009; Tauriainen et al., 2011). For example, mice on a 12-month CR diet exhibited increased SIRT3 levels in skeletal muscle compared to mice fed *ad libitum* (Palacios et al., 2009). In addition, *Sirt3* mRNA expression was increased in liver, skeletal muscle, and adipose tissue of mice on a 30% CR diet (Tauriainen et al., 2011). By contrast, a chronic high fat diet (HFD) resulted in suppression of SIRT3 and elevated global mitochondrial protein acetylation (Hirschey et al., 2011). Akin to CR, exercise training in mice increased SIRT3 protein expression in cardiac and triceps muscles (Palacios et al., 2009).

SIRT3 expression is also modulated during aging. Notably, elderly individuals exhibited a 50% reduction in peroxisome-proliferator activated receptor gamma coactivator 1-alpha (PGC-1α) and SIRT3 protein levels in skeletal muscle compared to young subjects, regardless of activity level (Joseph et al., 2012). By contrast, another study suggested exercise training could sustain equivalent levels of SIRT3 in skeletal muscle of both young and elderly individuals (Lanza et al., 2008). Additional studies are required to elucidate the mechanisms of these observations. One possible explanation for reduced SIRT3 levels during aging may be epigenetic regulation of the *Sirt3* gene promoter. Further investigation is required to determine the cause of SIRT3 downregulation during aging.

## SIRT3 IN MITOCHONDRIAL BIOGENESIS

Caloric restriction or exercise induce PGC-1α, a master regulator of genes involved in mitochondrial biogenesis, metabolism, suppression of ROS, and stress responses (Herzig et al., 2001; Yoon et al., 2001; Palacios et al., 2009). The importance of PGC-1α in these processes is underscored by observations of PGC-1α KO mice, which exhibit many defects including obesity, neurodegeneration, cardiomyopathy, and heightened sensitivity to ROS (Lin et al., 2004; St-Pierre et al., 2006).

Interestingly, PGC-1α may play a role in controlling *Sirt3* gene expression. A sequence motif in the *Sirt3* promoter is recognized by the estrogen related receptor-alpha (ERRα), an orphan nuclear receptor upregulated in CR (Ranhotra, 2009). PGC-1α mediates ERRα binding to this sequence motif in the *Sirt3* promoter and promotes *Sirt3* gene expression. Additionally, siRNA knockdown of PGC-1α reduces SIRT3 expression (Kong et al., 2010).

**FIGURE 1:**
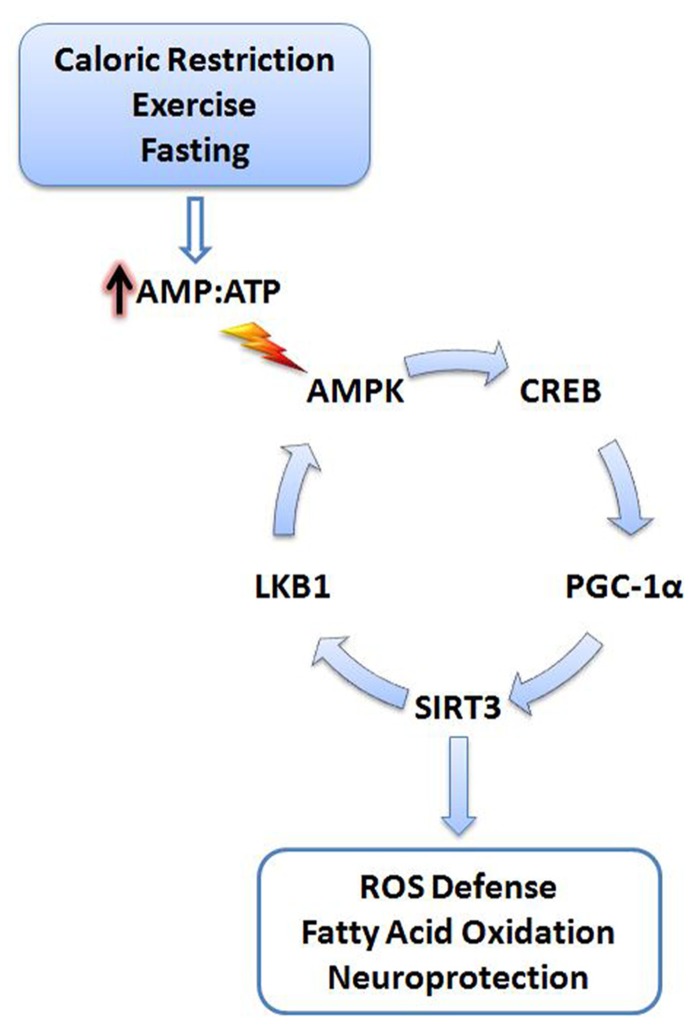
**Cellular stress activates SIRT3**. Energy deficits resulting from calorie restriction, exercise, and fasting cause the cellular AMP:ATP ratio to increase. Increased levels of AMP trigger activation of AMPK, initiating a signaling cascade promoting SIRT3 expression. SIRT3 promotes activation of antioxidant systems, fatty acid oxidation, and neuroprotection. A positive feedback mechanism is also initiated via the deacetylation and activation of LKB1 by SIRT3, further promoting activation of AMPK. AMPK, AMP-activated protein kinase; CREB, cyclic AMP response element-binding protein; PGC-1α, peroxisome proliferator-activated receptor gamma coactivator 1-alpha; LKB1, liver kinase B1; AMP, adenosine-5′-monophosphate; ATP, adenosine-5′-triphosphate.

SIRT3 may also upregulate PGC-1α through a positive feedback mechanism (**Figure 1**). SIRT3 deacetylates and activates liver kinase B1 (LKB1) in cardiomyocytes (Pillai et al., 2010). Active LKB1 phosphorylates and stimulates AMP-activated protein kinase (AMPK; Woods et al., 2003). As a result, activated AMPK phosphorylates cyclic AMP response element binding protein (CREB) leading to increased *PGC-1α* expression (Bergeron et al., 2001; Zong et al., 2002; Thomson et al., 2008). Moreover, AMPK can directly phosphorylate and activate PGC-1α, adding additional stimulatory mechanisms to this pathway (Jäger et al., 2007).

## NAD^+^ LEVELS GOVERN SIRT3 ACTIVITY

The rate-limiting factor of SIRT3 activity is the availability of NAD^+^, a required cofactor for functional deacetylase activity of the enzyme. Interestingly, the ratio of NAD^+^ to NADH is directly linked to cellular energy status, implicating SIRT3 as a key metabolic sensor (Kim et al., 2006). SIRT3 reacts with NAD^+^ and an acetylated protein substrate to produce nicotinamide (NAM), *O*-acetyl ADP ribose, and the deacetylated substrate (Onyango et al., 2002). Recycling of NAM to NAD^+^ is facilitated by nicotinamide phosphoribosyltransferase (Nampt), also known as visfatin (Rongvaux et al., 2002). Interestingly, Nampt expression increases upon fasting, thus further enhancing the activity of SIRT3 in mitochondria by almost doubling the local NAD^+^ concentration (Yang et al., 2007). By contrast, surplus energy, as is available under a HFD, reduces the NAD^+^/NADH ratio and thereby inadvertently decreases SIRT3 function (Kim et al., 2011a).

Aside from the sirtuins, a major consumer of NAD^+^ in the cell is poly (ADP-ribose) polymerase (PARP), an enzyme involved in DNA repair. PARP1-mediated NAD^+^ depletion is linked with neurodegeneration (Alano et al., 2004). Furthermore, activation of PARP1 in PD models contributes to neuronal apoptosis (Outeiro et al., 2007). However, PARP1 is located in the nucleus, which has its own separate pool of NAD^+^, and therefore, its deletion may not affect SIRT3 activity in mitochondria (Bai et al., 2011). Despite this compartmentalization, the activity of PARP1 may be indirectly regulated by the antioxidant function of SIRT3. Decreases in ROS caused by SIRT3 activity may reduce DNA damage and thereby reduce PARP1-mediated consumption of NAD^+^.

## SIRT3 ACTIVATES ANTIOXIDANT DEFENSES

Over 90% of cellular ROS is produced by mitochondria as electrons escape the ETC to combine with molecular O_2_ producing superoxide anions ($O_{2}^{•-}$; Finkel and Holbrook, 2000). Free radicals such as superoxide and H_2_O_2_ can cause considerable oxidative damage to proteins, lipids, and DNA, consequently expediting aging, cancer, and neurodegeneration (Balaban et al., 2005). Additionally, the detrimental effects of ROS become more pronounced with age due to the limits of cellular antioxidant defense systems. Mitochondrial ROS production increases in brains of aged mice (Sawada and Carlson, 1987). Similarly, as humans age there is a progressive trend toward a pro-oxidant state, perhaps as the cellular ROS defenses cannot keep pace with the steady, age-related increase in ROS production (Rebrin and Sohal, 2008). This notion is especially important for neurons that are highly susceptible to oxidative stress. Progressive ROS accumulation in neurons can result in exacerbated protein aggregation, cell death, and onset of neurodegeneration (Du et al., 2003).

The importance of ROS in aging is well illustrated by studies involving overexpression of antioxidant enzymes. In one particular study, overexpression of manganese superoxide dismutase (MnSOD) in *Drosophila* resulted in prolonged lifespan (Sun et al., 2002). Likewise, overexpression of catalase increased the lifespan of mice by 20% (Schriner et al., 2005). Interestingly, SIRT3 activity can reduce ROS levels by directly modulating key antioxidant enzymes, thereby acting as a shield against oxidative damage.

**FIGURE 2:**
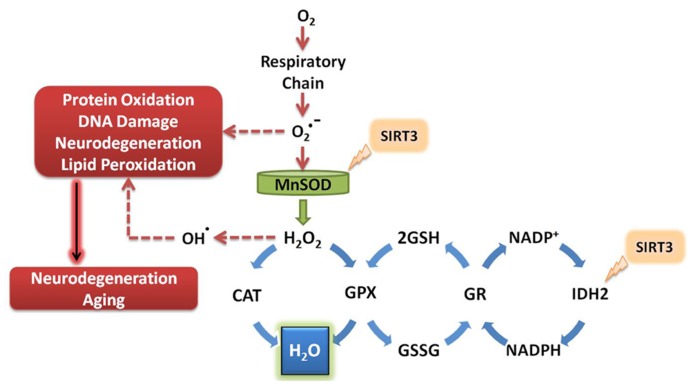
**SIRT3 promotes ROS defense systems.** SIRT3 deacetylates and activates MnSOD and IDH2, increasing their activity. MnSOD scavenges superoxide produced by the respiratory complexes, converting it to hydrogen peroxide which is further converted to water. Activation of IDH2 by SIRT3 increases its activity, thus producing more NADPH for use by glutathione reductase. GR converts oxidized glutathione to its reduced form, which is further used by GPX to convert the reactive hydrogen peroxide into water. O_2_, molecular oxygen; $O_{2}^{•-}$, superoxide; MnSOD, manganese superoxide dismutase; H_2_O_2_, hydrogen peroxide; CAT, catalase; GPX, glutathione peroxidase; GSH, reduced glutathione; GSSG, oxidized glutathione; GR, glutathione reductase; NADP^+^, nicotinamide adenine dinucleotide phosphate; NADPH, reduced nicotinamide adenine dinucleotide phosphate; IDH2, isocitrate dehydrogenase 2.

To date, the known antioxidant effects of SIRT3 are mediated by its interaction with MnSOD and isocitrate dehydrogenase 2 (IDH2; **Figure 2**). MnSOD is the primary mitochondrial antioxidant enzyme that converts $O_{2}^{•-}$ to H_2_O_2_, which is further converted to water by catalase (Spitz and Oberley, 1989). SIRT3 directly deacetylates MnSOD in mitochondria, significantly enhancing its ability to scavenge ROS (Qiu et al., 2010; Tao et al., 2010). SIRT3 also deacetylates IDH2, causing an increase in its activity (Mailloux et al., 2007; Someya et al., 2010; Yu et al., 2012). IDH2 is an enzyme of the tricarboxylic acid cycle which produces NADPH, a molecule implicated in the regeneration of antioxidants (**Figure 2**). Briefly, glutathione (GSH) acts as a major antioxidant in cells, reducing thiol groups of oxidized proteins and serving as mediator of oxidative stress responses (Berndt et al., 2007). Oxidized glutathione (GSSG) can be regenerated to GSH by glutathione reductase, an enzyme that is NADPH dependent (Anderson, 1998). Enhanced activity of IDH2 by SIRT3-mediated deacetylation produces increased levels of NADPH, which in turn can increase the activity of glutathione reductase to further facilitate regeneration of GSH from GSSG (**Figure 2**). During aging, oxidized glutathione accumulates and alters the mitochondrial GSH to GSSG ratio. Therefore, the ratio of GSH to GSSG can be used as marker for both cellular oxidative stress and aging (Rebrin et al., 2003).

The relevance of SIRT3 in antioxidant defense systems is evident in studies of mice under CR conditions. Accordingly, CR prevented age-related hearing loss in mice by increasing the mitochondrial GSH:GSSG ratio in wild-type mice, but not in SIRT3 KO mice (Someya et al., 2010). In another study, CR led to decreased ROS and increased cell survival by SIRT3-mediated deacetylation and activation of MnSOD (Qiu et al., 2010).

Aging and neurodegeneration are linked with increased oxidative stress and mitochondrial DNA (mtDNA) mutations. Transgenic mice expressing a proofreading-deficient mitochondrial polymerase gamma (Polγ), also known as mutator mice, exhibit increased mtDNA mutation rates, mitochondrial dysfunction, multisystem degeneration, and premature aging (Trifunovic et al., 2004). Remarkably, endurance exercise of the mice was able to entirely abolish the accumulation of mtDNA mutations and the premature aging phenotype (Safdar et al., 2011). The underlying mechanisms how exercise eradicates the accumulation of mtDNA mutations in these mice remain unknown. It is possible that exercise activates PGC1-α and SIRT3. Increased SIRT3 then lowers ROS-mediated mtDNA damage. Consistent with this idea, SIRT3 was recently found to directly deacetylate 8-oxoguanine-DNA glycosylase 1 (OGG1), a base excision repair enzyme located in both the nuclear and mitochondrial compartments. SIRT3 deacetylates and stabilizes OGG1, thereby promoting its capacity to repair mtDNA (Cheng et al., 2013). Furthermore, aged SIRT3 null mice exhibit increased oxidative stress and loss of mtDNA copies (Kim et al., 2010; Someya et al., 2010). It would be interesting to test in future experiments whether SIRT3 deletion in Polγ mutant mice abolishes the exercise-mediated protective effects against mtDNA mutations and premature aging.

In addition to increasing the activities of antioxidant systems such as MnSOD and IDH2, SIRT3 might also promote the transcription of oxidative stress response genes. Members of the fork head box subgroup O (FOXO) transcription factors regulate cell metabolism and the response to oxidative stress (Burgering and Kops, 2002; Accili and Arden, 2004). SIRT3 binds FoxO3a and promotes the transcription of *catalase* and *MnSOD* (Jacobs et al., 2008). In mouse cardiomyocytes SIRT3 overexpression elevates the mRNA levels of both *MnSOD* and *catalase* (Sundaresan et al., 2009). Additionally, in response to glucose restriction, FoxO3a translocates to mitochondria via an AMPK-dependent pathway. Within the mitochondrial matrix, SIRT3 deacetylates FoxO3a, allowing it to bind to mtDNA. Together, SIRT3 and FoxO3a are recruited with RNA polymerase to mtDNA and promote the upregulation of all 13 mitochondrial-encoded genes. As a result, an increase in mitochondrial respiration is observed, thus linking the AMPK–FoxO3a–SIRT3 pathway to the beneficial effects of CR in mammals (Peserico et al., 2013). Interestingly, the *Foxo3a* locus has previously been shown to be associated with longevity, underscoring its potential relevance in human aging (Willcox et al., 2008).

## MITOHORMESIS

Numerous studies have documented the deleterious effects of oxidative stress on nucleic acids, proteins, and lipids; however, beneficial effects of ROS have also been reported. Therefore, lowering ROS with antioxidants may not always be advantageous and may, contrary to the general belief, accelerate as opposed to slow aging. For example, administration of antioxidant vitamin C and vitamin E severely impairs the insulin-sensitizing and beneficial effects of exercise in humans (Ristow et al., 2009). The exercise-induced expression of SOD1, MnSOD, and glutathione peroxidase 1 were prevented by these antioxidants (Gomez-Cabrera et al., 2008; Ristow et al., 2009). Thus, ROS can have both positive and negative effects on human health. How can these seemingly contracting observations be reconciled? An explanation might be found in studies of the mitohormesis.

**FIGURE 3:**
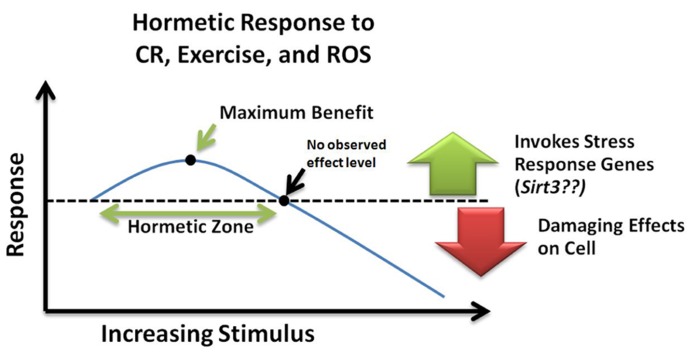
**Hormetic response curve**. Within the hormetic zone, mild or moderate doses of ROS, calorie restriction, and exercise may increase stress resistance and promote cell survival by invoking transcription of stress response genes such as *Sirt3*. Alternatively, high levels of cellular stress can cause damaging effects leading to cell death. NOEL, no observed effect level; CR, calorie restriction; ROS, reactive oxygen species.

Hormesis refers to the generally beneficial biological responses activated upon exposure to low levels of toxins or cellular stressors. Hormesis-stimulating compounds act by eliciting an adaptive stress response, which in turn conveys resistance to subsequent higher doses of the same agent (Kirkland, 2010). In one study, transient exposure of neurons to low levels of ceramide were protective against subsequent exposure to high levels of oxidative stress that would otherwise have induced cell death (Goodman and Mattson, 1996). Likewise, it can be speculated that the benefits of CR and exercise involve induction of a hormetic response (Kouda and Iki, 2010). Furthermore, protective stress response genes such as SIRT3 may be part of this hormetic response. However, the amount of agent required to produce a beneficial hormetic response may vary for each person depending on their susceptibility to that particular agent and might depend on genetic and epigenetic factors as well as age. Such a response can be visualized with an inverted U-curve or J-curve (**Figure 3**) specific to each stressor (Calabrese et al., 2012). These concepts of “preconditioning” or hormesis represent a helpful role for ROS promotion of a mitochondrial survival of the fittest. Low levels of ROS help select for healthy, resilient mitochondria, while inefficient, suboptimal mitochondria can be removed (Tapia, 2006).

## SIRT3 IN ENERGY METABOLISM

In the transition from the fed to fasted state many metabolic changes occur. Carbohydrate utilization in the liver ceases and fatty acid oxidation is upregulated resulting in production of acetyl-CoA (McGarry and Foster, 1980). Acetyl-CoA generated in the liver is then converted to acetoacetate, β-hydroxybutyrate, and acetone through ketogenesis in mitochondria. Ketone bodies released into the bloodstream can then be used for energy by both heart and brain tissue during starvation conditions (Laffel, 1999). Interestingly, human subjects on a short-term ketogenic diet exhibited elevated oxidative metabolism and improved ROS defense (Nazarewicz et al., 2007).

**FIGURE 4:**
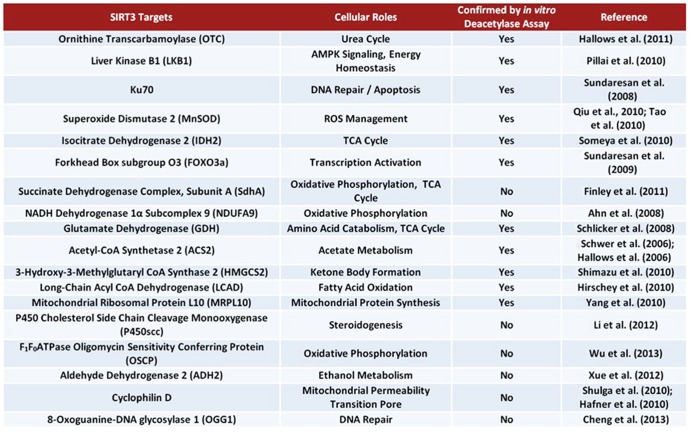
**Bona fide and suggested SIRT3 substrates**.

In response to low caloric intake, SIRT3 activates a vast array of proteins (**Figure 4**) associated with the Krebs cycle (tricarboxylic acid cycle), fatty acid oxidation, amino acid metabolism, and the ETC. To promote Krebs cycle activity, SIRT3 deacetylates and activates both IDH2 and succinate dehydrogenase A (SdhA). Interestingly, SdhA is also a component of the ETC, thus suggesting SIRT3 plays a role in increased respiratory complex II activity. In one study, SIRT3 KO mice exhibited a 30% reduction in complex II activity in BAT (Finley et al., 2011). SIRT3 also deacetylates NDUFA9, a subunit of NADH dehydrogenase (complex I), further enhancing ETC activity. In support of this idea, SIRT3 KO mice exhibited less complex I respiration activity. Furthermore, *Sirt3*^(-/-)^ mouse embryonic fibroblast (MEF) cells exhibited a 30% reduction in ATP levels (Ahn et al., 2008).

The use of amino acids for energy also becomes important during starvation conditions. SIRT3 deacetylates and activates glutamate dehydrogenase (GDH), promoting the conversion of glutamate to α-ketoglutarate, which can enter the Krebs cycle (Schlicker et al., 2008; Verdin et al., 2010; Dong et al., 2012). Additionally, SIRT3 deacetylates and activates ornithine transcarbamoylase (OTC), a critical enzyme in the urea cycle. Activation of the urea cycle could aid in amino acid catabolism during fasting conditions (Hallows et al., 2011). Remarkably, *Sirt3*^(-/-)^ MEFs exhibit accumulation of many amino acids, indicating a defect in amino acid catabolism (Hebert et al., 2013).

The large amount of acetate released into the blood stream by liver cells under fasting conditions is utilized mainly by the heart and skeletal muscle. These tissues express acetyl-CoA synthetase 2 (ACS2), an enzyme which catalyzes the ligation of acetate and CoA to form acetyl-CoA for use in energy production (Fujino et al., 2001). SIRT3 directly deacetylates and activates ACS2 in mitochondria, thereby promoting this process (Hallows et al., 2006; Schwer et al., 2006). Ketone body formation is also regulated by SIRT3 during fasting. SIRT3 deacetylates 3-hydroxy-3-methylglutaryl CoA synthase 2 (HMGCS2), elevating its activity and enhancing β-hydroxybutyrate production (Shimazu et al., 2010). Additionally, SIRT3 promotes the use of triacylglycerols in the liver by deacetylating and activating long-chain acyl co-enzyme A dehydrogenase (LCAD). Interestingly, mice lacking *Sirt3* exhibit accumulation of fatty acid oxidation intermediates and triacylglycerols in the liver (Hirschey et al., 2010).

The metabolic role of SIRT3 has also been proposed to extend to processes such as regulation of mitochondrial protein synthesis, steroidogenesis, and ATP synthesis. One report found that SIRT3 may associate with the mitochondrial ribosomal subunit MRPL10, thus implicating SIRT3 in regulation of mitochondrial protein synthesis (Yang et al., 2010). An interaction between SIRT3 and P450 cholesterol side chain cleavage monooxygenase (P450scc) has also been suggested. Specifically, overexpression of SIRT3 may stabilize P450scc by deacetylation, potentially connecting SIRT3 with steroidogenesis (Li et al., 2012). Additionally, potential regulation of the F_1_F_0_ATPase by SIRT3 has been proposed. A recent study demonstrated that SIRT3 binds to the oligomycin sensitivity conferring protein (OSCP), a subunit of the mitochondrial ATP synthase (Wu et al., 2013).

SIRT3 may also play a role in the response to consumption of ethanol. The oxidative metabolism of acetaldehyde (derived from ethanol) is facilitated by mitochondrial aldehyde dehydrogenase 2 (ALDH2), which is also an NAD^+^-dependent enzyme (Marchitti et al., 2008). Interestingly, significant acetylation of mitochondrial proteins is observed in liver tissues of mice fed high ethanol diets (Fritz et al., 2012). Remarkably, 30 min after acute ethanol treatment of human aortic endothelial cells (HAECs), the mitochondrial NAD^+^/NADH ratio decreased by 65%, thus potentially limiting the activities of NAD^+^-dependent enzymes such as SIRT3. Additionally, a decrease in the acetylation state of ALDH2 upon overexpression of SIRT3 suggests ALDH2 is a target of SIRT3; however, additional studies must be performed to confirm this interaction (Xue et al., 2012).

Numerous reports suggest that SIRT3 may act to prevent metabolic maladies such as insulin resistance, metabolic syndrome, and obesity. Mice fed a chronic HFD exhibit elevated global mitochondrial protein acetylation as a result of suppression of SIRT3 (Hirschey et al., 2011). This global acetylation of the mitochondrial proteome may play a role in HFD-induced liver injury (Choudhury et al., 2011). Moreover, SIRT3 KO mice exhibited impaired insulin action due to increased ROS accumulation (Jing et al., 2011). Finally, PGC-1α KO mice exhibited many metabolic defects including obesity, cardiomyopathy, and neurodegeneration (Lin et al., 2004). PGC-1α-mediated upregulation of SIRT3 may play a role in preventing such illnesses.

## SIRT3 IN NEUROPROTECTION

The large ATP requirements of neurons predispose them to insults that result in energy depletion, including DNA damage, excitotoxicity, and oxidative stress (Sokoloff, 1981; Du et al., 2003). Within neurons, mitochondria are the main sources of ROS and energy production, suggesting these specialized organelles are critical mediators of age-related diseases such as neurodegeneration (Singh, 2006). Surprisingly, CR reduces neurodegeneration in animal models of both PD and AD, possibly via upregulation of SIRT3 (Zhu et al., 1999; Mattson, 2000). Furthermore, overexpression of SIRT3 has been shown to significantly increase neuronal lifespan (Weir et al., 2012). The antioxidant and metabolic effects mediated by SIRT3 suggest a potential neuroprotective role through improved mitochondrial function, which subsequently results in increased neuronal survival and reduced aging effects.

The direct causes of many forms of neurodegeneration remain unknown, though insults or agents resulting in neuronal cell death are likely to play a key role. Studies in aged rat brain have revealed an age-dependent increase in both mitochondrial ROS production and cytosolic Ca^2+^ levels (Sawada and Carlson, 1987; Xiong et al., 2002; Toescu and Vreugdenhil, 2010). Interestingly, increased levels of ROS and Ca^2+^ trigger mitochondrial permeability transition pore (mtPTP) formation, an event which can lead to apoptosis and trigger neurodegeneration (Du and Yan, 2010). Briefly, the mtPTP includes the voltage-dependent anion channel (VDAC), adenine nucleotide translocator (ANT), and cyclophilin D (CypD). In response to ROS and increased Ca^2+^, binding of CypD to ANT initiates formation of a tunnel-like structure, which connects the mitochondrial matrix to the cytosol resulting in the rapid flow of NAD^+^ from the mitochondria to the cytosol (Lemasters et al., 2009). Within the cytosol, NAD^+^ is quickly hydrolyzed by multiple NADases to yield ADP-ribose and NAM (Zhang et al., 1995; Bernardi, 1999). The frequency of mtPTP formation may result in the destruction of defective mitochondria by autophagy or possibly even cell death via apoptosis (Kim et al., 2007). Recent studies suggest SIRT3 may be able to suppress mtPTP formation during aging. In response to CR, SIRT3 is upregulated and directly deacetylates CypD, preventing its association with ANT and therefore blocking mtPTP formation (Hafner et al., 2010; Shulga et al., 2010). Additionally, interaction between CypD and amyloid-β in mitochondria of AD patients has been reported. Such an interaction caused increased oxidative stress and increased mtPTP opening, triggering neurodegeneration (Du and Yan, 2010). Upregulation of SIRT3 may be able to prevent or delay this process, conveying a neuroprotective effect in AD.

Recent observations also hint at additional neuroprotective effects of SIRT3 involving regulation of mitochondrial dynamics. A defective mitochondrial fission and fusion balance affects mitochondrial transport and function, potentially leading to synaptic dysfunction and neurodegeneration (Knott et al., 2008). In fact, mutations in the mitochondrial fusion protein OPA1 cause dominant optic atrophy, thereby linking mitochondrial dynamics with neuronal functionality (Seo et al., 2010). In a recent study, SIRT3 was able to rescue the mitochondrial fragmentation associated with a model of amyotrophic lateral sclerosis. Specifically, spinal cord motor neurons transfected with SOD1^G93A^ displayed an increase in round fragmented mitochondria in addition to defects in bi-directional axonal transport and increased cell death. However, co-expression with either SIRT3 or PGC-1α was able to rescue SOD1^G93A^-induced mitochondrial fragmentation and improve cell survival (Song et al., 2012). Interestingly, PGC-1α directly regulates mitochondrial dynamics by increasing MFN2 expression (Soriano et al., 2006). It may be that by inducing PGC-1α, ROS is decreased by the antioxidant stimulating abilities of SIRT3, while mitochondrial fusion is increased by MFN2. Additionally, PGC-1α null mice are more sensitive to the neurodegenerative effects of ROS, further identifying itself and its target genes such as *Sirt3* as potential mediators of neuroprotection (St-Pierre et al., 2006).

In neurons, increased SIRT3 expression has also been reported in response to oxidative stress (Kim et al., 2011b). Furthermore, oxidative stress has been shown to upregulate β-secretase activity, an enzyme associated with AD (Tamagno et al., 2002). In a study utilizing a mouse model of AD, *Sirt3* mRNA upregulation mirrored spatiotemporal amyloid-β deposition. Additionally, Sirt3 mRNA was found to be increased in human post-mortem cortex samples of AD patients (Weir et al., 2012). In this case, it may be that upregulation of SIRT3 is a compensatory mechanism in neurons to attempt to protect against the increased oxidative stress that accompanies AD development and progression.

The ability of CR to induce SIRT3 expression has been well documented. Additional stimulators of the Sirtuins have also been proposed, such as resveratrol, a polyphenol found in red wine. However, the effect of resveratrol on Sirtuin expression remains controversial. In one study, mice on a 30% CR diet exhibited increased *Sirt3* mRNA levels, while treatment with resveratrol did not affect *Sirt3* expression levels. In light of this finding, resveratrol may be ineffective in mimicking CR-mediated health benefits (Tauriainen et al., 2011). However, not all hope is lost for the polyphenol compounds. A recent report found that a resveratrol derivative, trans-(-)-ε-viniferin, is able to increase SIRT3 expression and provide protection in cell models of Huntington’s disease (HD). Specifically, viniferin treatment of striatal precursor cells overexpressing mutant huntingtin resulted in increased SIRT3 expression, increased the NAD^+^/NADH ratio, reduced intracellular ROS accumulation, and decreased acetylated MnSOD levels. Additionally, treatment with viniferin increased levels of activated AMPK and decreased acetylated LKB1, effects which were shown to require the presence of SIRT3. Thus, *Sirt3* is required for viniferin-mediated neuroprotection in HD models (Fu et al., 2012).

## CONCLUSIONS AND FUTURE PERSPECTIVES

The beneficial effects of SIRT3 in regulating metabolism and activating antioxidant defense systems in response to CR and exercise is apparent. Additionally, while mechanisms by which SIRT3 can provide neuroprotection are better understood, there are still some discrepancies among studies that have not been accounted for. To date, the majority of studies acknowledge the strict localization of active SIRT3 to mitochondria. Despite this, SIRT3 activity has been reported in the nucleus where it plays a role in associating with Ku-70 to help promote cell survival (Sundaresan et al., 2008). The discovery of a splice variant in mice that lacks a MTS might help explain such observations, but further experiments could help settle any disputes over SIRT3 subcellular localization. Additionally, short-term and long-term CR appear to have varying effects on SIRT3 expression (Jing et al., 2011). What should be the recommended level of CR to maximally induce SIRT3 expression? Furthermore, the decrease in SIRT3 expression with age cannot be explained. What transcriptional regulators affect expression of SIRT3 during aging and how do these factors get turned on or off over time? These questions remain unanswered and require further investigation. Finally, recent global mitochondrial protein acetylome studies have been performed, potentially identifying a myriad of exciting SIRT3 substrates that are not yet known (Hebert et al., 2013). Identification of additional bona fide SIRT3 targets will help solidify our understanding of the role of SIRT3 in neuroprotection and longevity.

It has been proposed that excessive energy intake can expose humans to oxidative consequences during the fourth and fifth decade of life, potentially leading to cognitive decline during later years (Debette et al., 2011). Conversely, CR can promote healthy aging and neuroprotection via the actions of SIRT3. However, the eating habits of the majority of Western civilization are far from conducive for induction of optimal SIRT3 expression. For this reason, molecular mimetics of CR are needed to substitute for an actual decrease in food intake. In addition, novel agents that can induce a beneficial hormetic response to promote the upregulation of stress response genes such as *Sirt3* may also be of great therapeutic value, providing a means for enhancing neuroprotection and healthy aging.

## Conflict of Interest Statement

The authors declare that the research was conducted in the absence of any commercial or financial relationships that could be construed as a potential conflict of interest.
